# Recurrent Neural Networks for Mexican Sign Language Interpretation in Healthcare Services

**DOI:** 10.3390/s26010027

**Published:** 2025-12-19

**Authors:** Armando de Jesús Becerril-Carrillo, Héctor Julián Selley-Rojas, Elizabeth Guevara-Martínez

**Affiliations:** Faculty of Engineering, Universidad Anáhuac México, Avenida Universidad Anáhuac 46, Col. Lomas Anáhuac, Huixquilucan 52786, Estado de Mexico, Mexico; armando.becerrilca@anahuac.mx (A.d.J.B.-C.); hector.selley@anahuac.mx (H.J.S.-R.)

**Keywords:** artificial intelligence, Mexican Sign Language, computer vision, precision, recall, LSTM, GRU, data augmentation

## Abstract

In Mexico, the Deaf community faces persistent communication barriers that restrict their integration and access to essential services, particularly in healthcare. Even though approximately two million individuals use Mexican Sign Language (MSL) as their primary form of communication, technological tools for supporting effective interaction remain limited. While recent research in sign-language recognition has led to important advances for several languages, work focused on MSL, particularly for healthcare scenarios, remains scarce. To address this gap, this study introduces a health-oriented dataset of 150 signs, with 800 synthetic video sequences per word, totaling more than 35 GB of data. This dataset was used to train recurrent neural networks with regularization and data augmentation. The best configuration achieved a maximum precision of 98.36% in isolated sign classification, minimizing false positives, which is an essential requirement in clinical applications. Beyond isolated recognition, the main contribution of this study is its exploratory evaluation of sequential narrative inference in MSL. Using short scripted narratives, the system achieved a global sequential recall of 45.45% under a realistic evaluation protocol that enforces temporal alignment. These results highlight both the potential of recurrent architectures in generalizing from isolated gestures to structured sequences and the substantial challenges posed by continuous signing, co-articulation, and signer-specific variation. While not intended for clinical deployment, the methodology, dataset, and open-source implementation presented here establish a reproducible baseline for future research. This work provides initial evidence, tools, and insights to support the long-term development of accessible technologies for the Deaf community in Mexico.

## 1. Introduction

Communication is an essential component of human interaction, enabling social bond building, access to education, and integration into professional life. In Mexico, according to data from the Secretaría de Salud (Mexican Ministry of Health), approximately 2.3 million people live with some form of hearing impairment, more than 50% of whom are over 60 years old; slightly more than 34% are between 30 and 59 years old and about 2% are children [1]. For this population, Mexican Sign Language (MSL) represents not only a means of communication but also a central element of their cultural identity. However, the lack of knowledge about MSL among the hearing population, and especially among personnel in critical sectors such as healthcare, remains an obstacle to effective societal inclusion.

At a global level, multiple international organizations have documented the persistent communication barriers faced by Deaf communities when accessing health information and services. In its 2019–2023 Global Report, the World Federation of the Deaf (WFD) highlights that, during the COVID-19 pandemic, Deaf individuals around the world experienced systemic exclusion due to the absence of sign language interpretation in official health communications, press conferences, and remote medical services. This lack of accessibility led the WFD and the World Association of Sign Language Interpreters to issue multiple policy statements and collaborate with the World Health Organization (WHO) to revise disability-related guidelines, requiring national governments to ensure access to public communications in national sign languages during health emergencies [2].

These forms of exclusion have also been observed at a regional level. The Economic Commission for Latin America and the Caribbean (ECLAC) has reported that Deaf persons in Latin America have some of the lowest levels of effective access to healthcare among disability groups. ECLAC identifies persistent communication barriers, a lack of accessible information in sign languages, and long-standing structural inequalities as primary contributors to this gap [3].

In Mexico, national human rights institutions and academic studies have reinforced this diagnosis. In a 2019 communication, the Comisión Nacional de los Derechos Humanos (CNDH) stated that persons with disabilities continue to face significant and persistent lags in accessibility, access to healthcare, education, work, independent living, and political participation, despite the existence of a legal and institutional framework intended to guarantee their rights [4]. More recently, a CNDH special report on accessibility and health services documented that the Ministry of Health has established a strategic goal to ensure that 100% of high-concentration public hospitals provide access to certified MSL interpreters. However, the baseline for this indicator remains effectively at 0%, revealing a substantial gap between policy commitments and actual implementation [5].

An academic analysis by the National Autonomous University of Mexico highlights that communication barriers in healthcare disproportionately affect persons with disabilities, including the Deaf community. The report emphasizes that integrating MSL interpreters and accessible communication tools is essential in guaranteeing dignified and non-discriminatory medical care; an absence of these measures leads to compromised clinical interactions, a lack of informed consent, and reduced quality of care [6]. For example, during the pandemic, the Mexican Institute for Disability made a national directory of MSL interpreters publicly available. They listed only 56 contacts nationwide, highlighting the critical shortage of interpreting services [7]. Together, these global, regional, and national findings underscore the urgent need for technological systems that can support communication in healthcare settings, particularly in environments where interpreter availability is limited or nonexistent.

To address these challenges, recent reviews highlight rapid progress in sign language (SL) technologies across multiple regions and languages, such as American Sign Language (ASL), Arabic Sign Language (ArSL), Chinese SL, Algerian SL, Kannada SL, Turkish SL, and others. These surveys highlight several limitations, including the scarcity of annotated datasets, challenges associated with capturing temporal dependencies in continuous signing, and the lack of domain-specific resources for applications such as healthcare [8,9,10]. In addition, with respect to the techniques applied, combinations of Convolutional Neural Networks (CNNs) and Recurrent Neural Networks (RNNs), as well as attention-based models, remain predominant. Also, pose-estimation methods with RNN have demonstrated comparable effectiveness [11,12].

In clinical contexts, researchers have proposed a variety of architectures to improve doctor–patient communication. Existing solutions include CNNs, such as 1D-CNN systems for Algerian SL medical terms [13], hybrid CNN–Transformer models for Kannada SL [14], and CNN–LSTM (Long Short-Term Memory) frameworks with attention trained on public datasets [15]. Among these, the lightweight CNN-based “Heart-Speaker” device [16] stands out as a practical prototype, recognizing 19 Mandarin Chinese SL gestures using MobileNet-YOLOv3 and enabling interaction between clinicians and deaf patients. These proposals support only limited vocabularies, and continuous recognition remains particularly challenging.

Even advanced temporal models—such as Temporal Convolutional Network (TCN), Bidirectional Long Short-Term Memory (BiLSTM), and CNN–LSTM hybrids—continue to struggle with coarticulation, movement epenthesis, and signer variability. Current continuous-signing work, including studies on ArSL, generally targets constrained sentence templates rather than spontaneous narrative input [17]. All these advances clearly demonstrate the promise of these technologies for addressing the communication barriers faced by deaf individuals. However, healthcare-focused datasets for MSL are not yet available, and prior studies have not examined narrative or continuous-sign recognition in this language.

This paper presents an exploratory and methodologically structured study on automated MSL recognition, focusing on vocabulary relevant to healthcare communication. The aim is not clinical deployment but to assess the feasibility of sequential MSL interpretation models that could eventually support communication in remote or low-resource medical contexts. This study is therefore a pilot investigation designed to provide empirical evidence for future large-scale research.

A domain-specific dataset was developed comprising 150 medical and functional signs, each with 800 video sequences, resulting in more than 35 GB of curated data. The recordings were produced by a single native signer, a design choice that ensures methodological consistency but limits the model’s generalization to broader populations. This constraint is explicitly acknowledged as part of the exploratory nature of the study and serves as motivation for future expansion to include multi-signer and multi-environment datasets.

It is also important to note that the sequential evaluations conducted in this study were based on synthetic narrative videos generated from the same native signer used in the training data. This strategy allowed us to emulate short, domain-relevant scenarios and verify end-to-end functionality under controlled conditions.

The study evaluates RNN architectures, specifically LSTM and GRU (Gated Recurrent Unit) models, optimized using dropout, batch normalization, and early stopping. Under the controlled conditions of this pilot dataset, the models achieved validation accuracies above 99% for isolated sign recognition.

To explore preliminary robustness, we applied an extensive data augmentation strategy incorporating geometric transformations, temporal variation, and illumination changes, approximating some real-world variability while acknowledging that these manipulations cannot replace true linguistic and inter-signer diversity.

Finally, the system was evaluated using short, domain-relevant narrative test cases to examine its ability to handle sequential MSL interpretation. These narrative sequences were generated synthetically from the same native signer as the isolated-sign dataset. This approach allowed us to emulate simple medical scenarios under controlled and internally consistent conditions. Nevertheless, it also implies that the evaluation reflects signer-dependent representation rather than a generalizable multi-signer model. The system achieved a global sequential recall of 45.45%, revealing that, while RNNs perform well for isolated signs, they experience significant degradation when interpreting continuous sequences—an expected outcome in pilot-scale sign language research. These findings underscore the need to explore more advanced temporal and multimodal architectures capable of capturing grammatical and semantic dependencies in MSL.

Despite the controlled and signer-dependent nature of synthetic narrative evaluations, this study establishes a reproducible methodological baseline for future research on MSL video processing, synthetic data generation, and preliminary sequential inference. The proposed framework is structured to support future expansion—namely the inclusion of multi-signer datasets and more diverse recording environments—and potential applications in remote communication, tele-triage or assistive communication tools.

The key contributions of this research can be summarized as follows:

- Curated and standardized dataset: The first healthcare-oriented MSL dataset of 150 signs with 800 samples each (over 120,000 samples and 35 GB of data), processed using 226 MediaPipe (v0.9.2.1) keypoints.
- Advanced synthetic data augmentation: A three-fold strategy—temporal, geometric, and illumination-based—yielding 799 synthetic videos per class.
- Sequential narrative inference: A narrative-level evaluation framework using six medical test cases.

The remainder of this paper is organized as follows: Section 2 reviews related work and introduces the relevant background concepts. Section 3 describes the proposed methodology, including the stages of MSL understanding, data collection and processing, as well as the training and evaluation procedures. Section 4 presents and discusses the experimental results, while Section 5 introduces the proposed model for MSL Grammar Inference, evaluates its performance, and discusses the findings. Finally, Section 6 concludes the paper and outlines directions for future work.

## 2. Related Work

Driven by advancements in artificial intelligence and computer vision, the scientific community has shown a growing interest in the automatic MSL recognition in recent decades. These technologies have demonstrated significant potential for facilitating communication between deaf and hearing individuals, particularly in contexts where the presence of human interpreters is not always feasible.

To address this challenge, various studies have explored approaches based on RNNs, CNNs, and hybrid architectures. For example, Martínez-Sánchez et al. (2023) developed the MX-ITESO-100 dataset, consisting of 100 gestures and 5000 videos captured in uncontrolled conditions, allowing for deep learning model evaluation in realistic contexts [18]. Borges-Galindo et al. (2024) implemented RNN models such as LSTM, GRU, and BiLSTM, achieving 93% accuracy in offline tests [19]. Sánchez-Vicinaiz et al. (2024) used a combination of CNN and MediaPipe for finger-spelling recognition, achieving an accuracy of 83.63% [20]. Finally, Mejía-Peréz et al. (2022) employed an RGB-D camera along with LSTM and GRU models to capture 3D sequences, obtaining an accuracy of 97% on clean data and 90% on noisy data [21]. It is worth mentioning that other studies have focused on the creation of sign language databases. For example, Espejel et al. (2024) introduced an extensive dataset of 249 words in MSL, comprising 31,442 images extracted from videos [22]. The dataset ensures uniformity in background and clothing to enhance contrast in hand and face detection [22].

Although these studies represent significant advancements, they share some limitations: most focus on general vocabularies or static gestures, lacking thematic specialization; additionally, few incorporate data augmentation techniques that enhance the robustness and generalization of models in real conditions. In this sense, existing approaches face challenges in terms of practical implementation in specific contexts such as the medical field, where lighting conditions, natural movement, and spontaneous gestures can vary widely.

To contextualize our research and highlight its contributions, Table 1 shows a detailed comparison between our approach and that of previously mentioned works. This comparison helps identify key aspects where our proposal offers improvements, such as the use of a specialized vocabulary, the volume and diversity of the dataset, the application of advanced augmentation techniques, and the design of multiple experimental configurations that optimize the recurrent architecture. Furthermore, unlike previous works focused on static sign recognition or finger-spelling, our approach introduces a health-specific vocabulary of 150 signs and evaluates both word-level classification and full narrative interpretation. While individual sign precision reaches 98.36% for the best model, the proposed system also achieves an average recall of 45.45% in reconstructing coherent sequences, highlighting its potential for dynamic, context-aware interpretation. This dual evaluation framework represents a significant step in advancing research on continuous MSL processing, providing a reproducible foundation for future sequence-level studies.

## 3. Methodology

**Figure 1:**
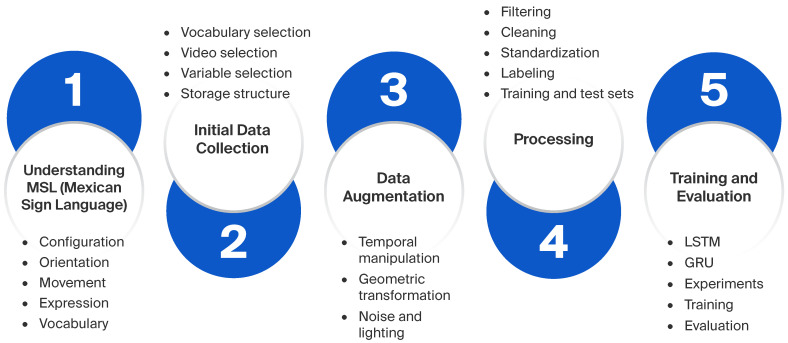
General diagram of the proposed system’s methodology.

Our methodology is structured into five fundamental phases, as represented in Figure 1, reflecting both the technical dimension and sociolinguistic understanding of MSL. Unlike other studies focused exclusively on computational aspects, this work recognizes that effective MSL interpretation requires a deep understanding of its grammar, cultural context, and everyday use, especially in sectors such as healthcare.

### 3.1. MSL Understanding

Before addressing any technical or engineering contributions, the first step of this research involved integral and committed MSL immersion. This initial phase aimed to not only acquire linguistic competencies but also gain an in-depth understanding of the cultural, linguistic, and social particularities of the Deaf community in Mexico.

To achieve this, specialized courses were taken with a native deaf teacher, offered by the online MSL school Rompiendo Muros [23], an institution recognized for its commitment to professional MSL teaching and its extensive experience and educational resources. This formative and experiential journey was invaluable, as it allowed for the internalization of not only the technical aspects of the language but also the generation of empathy with the user community, understanding their history and culture, as well as the daily communication challenges they face.

#### 3.1.1. Linguistic and Cultural Foundations of MSL

MSL is a natural and visual means of expression used by Deaf communities. As noted by J. Jiménez and A. Martín [24], sign languages are not only communication tools; they also reflect the cultural identity of the communities that use them. According to the Centro Universitario de Veracruz and Ana Luisa Solís González in their research “Real-Time Mexican Sign Language Recognition” [25], MSL consists of a series of gestural signs articulated with linguistic function, with a grammar as complex and structured as any spoken language. In Mexico, MSL is officially recognized as a national language, forming an integral part of the linguistic and cultural heritage of the Deaf community [26].

Studies by Stokoe [27] and Goldin-Meadow and Brentari [28] have shown that sign languages, including MSL, have a complete and autonomous grammatical structure, with their own lexicon, syntax, and morphology. This linguistic richness is reflected in MSL’s ability to convey abstract and complex ideas through visual–spatial parameters.

##### Common Misconceptions and Clarifications

There are frequent misconceptions about MSL and other sign languages. It is important to clarify that MSL is not “Spanish in signs” nor a direct translation of spoken Spanish. Moreover, there is no universal sign language, and MSL is not confined to mime, spontaneous gestures, or the exclusive use of finger-spelling.

##### Parameters and Grammar of MSL

The articulation of signs in MSL is based on five fundamental parameters, which allow for a precise and visual linguistic structure:

- Configuration: The shape adopted by the hands.
- Orientation: The direction in which the hands point.
- Space: The location at which the signs are executed in relation to the body.
- Movement: Any displacement performed by the hands.
- Expression: Facial and bodily expressions that accompany the sign.

Additionally, the sentential structure in MSL follows this characteristic logical and visual order:

Time – Place – Subject – Object – Verb – Question.

A crucial consequence of the grammatical structure of MSL is that its parameters cannot be interpreted independently, as manual and non-manual features operate simultaneously to form inseparable semantic units. Linguistic studies in sign languages have shown that identical manual configurations may correspond to different lexical meanings depending solely on facial expression or head movement, as exemplified by SKIN and RACIST signs in the French Sign Language, which share the same manual articulation but differ only in facial expression [29]. Similar phenomena are also observed in MSL, where affirmative and negative interpretations (e.g., RECORDAR vs. NO RECORDAR) may rely only on non-manual markers such as head movements. From a computational perspective, recent vision-based studies emphasize that removing articulators, including the face or body pose, may simplify classification tasks but undermines full semantic inference, particularly in continuous and narrative-level sign language understanding [30]. This linguistic characterization provided the methodological choices adopted in this study.

In addition, an immersion and understanding phase enabled the definition of an informed and strategic vocabulary of 150 signs for system training. Unlike other approaches that select gestures at random or for technical convenience, this research adopts an ethically grounded and user-community-centered approach. Vocabulary selection was primarily oriented toward the healthcare sector, given its relevance for improving communication in clinical, emergency, and primary care contexts.

Consequently, this first step not only laid the linguistic foundations of the project, but also aligned it with principles of inclusion and social relevance, both of which are fundamental pillars for the development of truly transformative technologies.

### 3.2. Data Collection

Vocabulary selection was a meticulous process that involved the identification of 150 key words from MSL, along with their corresponding sequences. This vocabulary was carefully designed to include both everyday terms and specialized concepts from the medical field, thus covering a representative and functional lexical spectrum.

To enrich this project, we established a collaboration with the online MSL school Rompiendo Muros [23]. This partnership allowed the production of a set of videos that accurately represent the selected vocabulary. The videos are of suitable quality and precision for this study and are summarized in Table 2.

This methodology ensured both technical quality and linguistic fidelity in the base material, both of which are fundamental aspects for the research objectives. An overview of the selected vocabulary is shown in Table 3.

**Figure 2:**
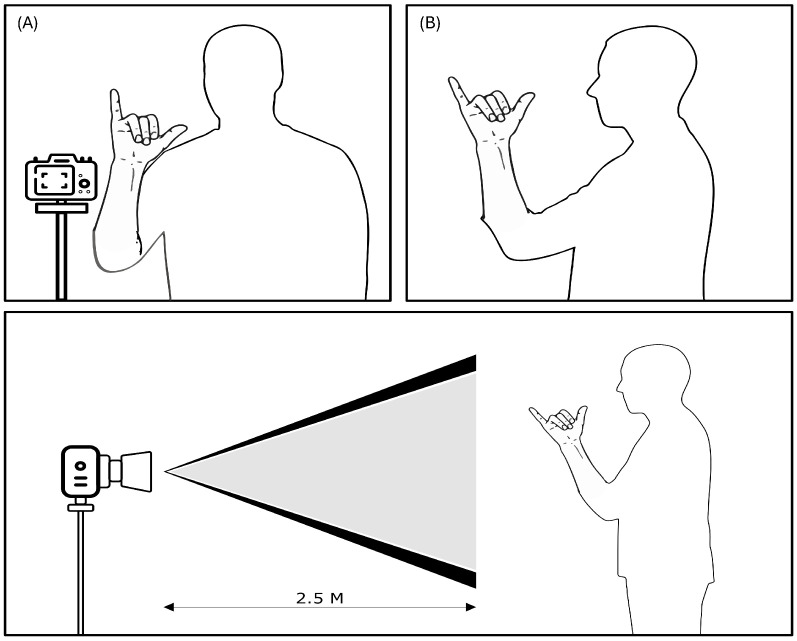
Setup used for recording the MSL corpus. A full HD camera was positioned at a distance of 2.5 m, capturing at 30 FPS. (**A**) shows the front view, (**B**) the side view, and the lower diagram illustrates the camera’s field of view.

Once the final set of 150 key words was defined—as selected from an initial corpus of over 1500 signs provided by [23]—the data collection and structuring process began. A base sample of 150 original videos—1 per word—was generated. These videos were recorded by a single native deaf teacher, controlling for consistency in lighting, background, and attire. This visual homogeneity was crucial to minimizing noise in subsequent analytical stages. The setup used for recording the MSL corpus is presented in Figure 2.

Each video was organized into independent folders and labeled according to the corresponding word, facilitating an automated processing workflow. The tool used was MediaPipe (v0.9.2.1) [31], a computer vision solution optimized for real-time tasks and compatible with TensorFlow (v2.13.0).

This tool offers a hand-tracking solution (MediaPipe Hands) that automatically generates 21 hand landmark points (wrist, metacarpals, phalanges, and fingertip). Since the hands are the main component in MSL, these 21 variables represent the key information extracted from the motion sequences in the dataset, playing a central role in sign analysis and recognition.

These interpretations are stored in a sequence of movements for each hand position and finger configuration, with all information processed and organized by variables. However, due to the various hand configurations and MSL’s numerous signs, both hands are considered. Therefore, 21 points are obtained each for the left and right hands, resulting in a total of 42 points for both. With 3 coordinates per point (X, Y, Z), this yields a total of 126 hand data variables.

**Figure 3:**
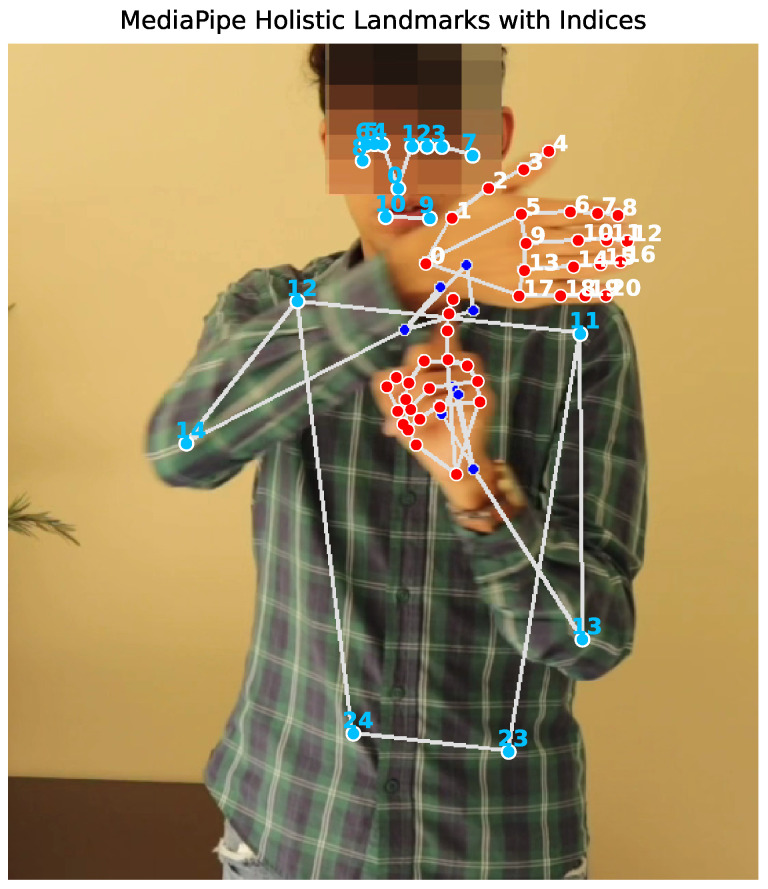
Schematic representation of the MediaPipe Holistic landmark indices used in this study. Blue dots indicate upper-body pose landmarks, while red dots correspond to hand landmarks. The hand model provides 21 landmarks per hand (42 total), and the pose model provides 25 upper-body landmarks (0–24), excluding all lower-body points, as leg and foot landmarks are not required for Mexican Sign Language representation (some landmarks may appear partially overlapped or occluded due to the 2D projection and visualization perspective).

Other variables that enrich the knowledge base are human pose key points, for which the MediaPipe Pose solution [32] is used. MediaPipe Pose provides full-body tracking with 33 reference points, including below the hip. However, because these points are not essential for MSL, a preliminary selection of pose points was performed, resulting in 25 points (up to the hip) being used as characteristic variables. Each of these points contains four values (X, Y, Z, visibility), and Figure 3 illustrates the spatial structure underlying the 230–dimensional feature vector extracted from each frame.

Table 4 presents a detailed breakdown of the categories and number of variables included in the research. As can be seen, these variables are distributed across different groups, including both general elements and specific components related to hand and pose information. This process effectively converts image sequences into numerical temporal data that RNN-based architectures can efficiently process.

### 3.3. Data Augmentation and Processing

Recognizing that MSL communication involves significant variations in speed, body orientation, and environmental conditions, we applied a set of advanced transformations to the original video to increase model robustness and generalization. The specific augmentation operations are summarized in Table 5.

These steps generated 799 synthetic videos for each of the 150 words, yielding a final set of 800 videos per word. Transformations were implemented through an automated Python pipeline with the moviepy library.

The effect of data augmentation can be expressed as:

(1) T(x)=R(θ)S(v)A(I)x

where T(x) is the total transformation of the original video, R(θ) is the rotation with $\theta \in [-10^{\circ },\;10^{\circ }]$, S(v) is the temporal scaling with v∈[0.85, 1.15] and A(I) is the illumination alteration via Gaussian noise.

These transformations enable the training of more resilient models under realistic clinical variability, consolidating this study’s engineering contribution.

In comparison with existing MSL dataset proposals [19,21,22], the present work expands the available vocabulary to 150 signs, provides 800 temporally aligned sequences per class through controlled data augmentation, and incorporates a lightweight temporal smoothing mechanism based on a 10-frame sliding window.

This combination yields high precision in isolated-sign recognition, enabling the preliminary reconstruction of short sign sequences and offering an initial path towards scalable sequence modeling in MSL.

Once the original video sequences were collected and the augmented versions generated through synthetic transformations, we processed, labeled, and standardized the entire dataset to create a uniform structure suitable for efficient and reliable integration into deep learning architectures.

As previously mentioned, the initial processing was performed using the MediaPipe framework, with each processed frame converted into a structured feature vector composed of 230 variables. All temporal sequences were standardized and stored in CSV format, forming the foundation of the publicly released MSL-150 Mexican Sign Language Dataset, available on Zenodo  [33].

**Figure 4:**
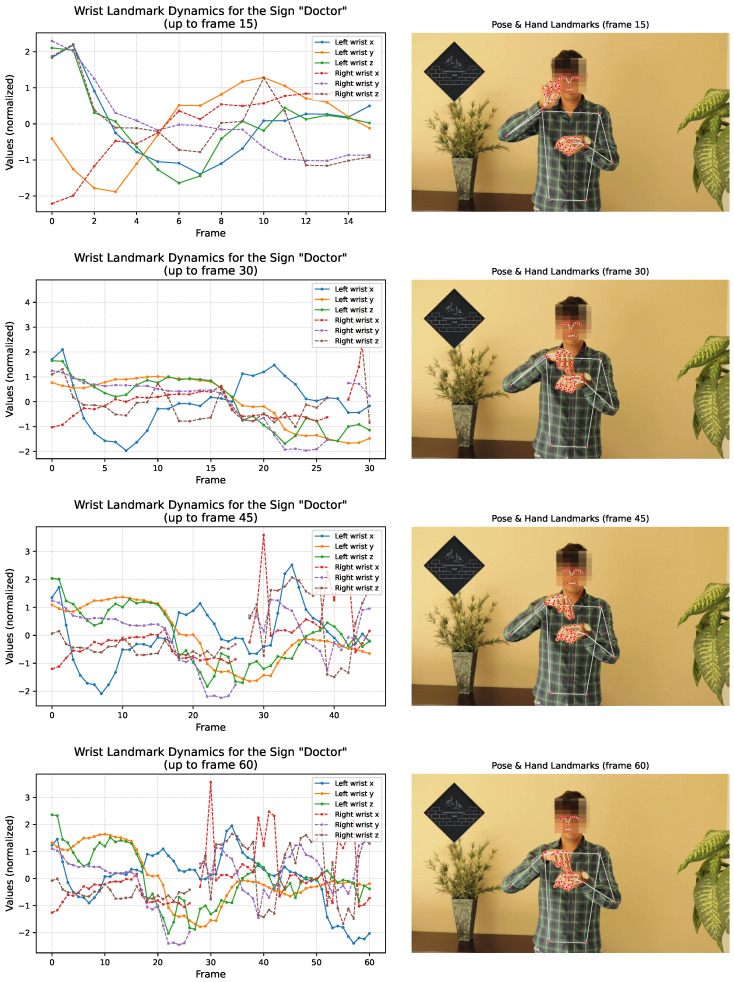
Sequential visualization of the “Doctor” sign. Each panel shows (**left**) the normalized wrist landmark dynamics up to frames 15, 30, 45, and 60, and (**right**) the corresponding annotated MediaPipe frame. This illustrates how temporal information is encoded and directly linked to the extracted pose and hand landmarks.

To complement this description, we additionally provide a visual illustration of how the temporal sequences for different signs evolve over time. Figure 4 presents a set of representative frames extracted from the raw videos, together with their corresponding MediaPipe landmarks (hands and upper body) and normalized wrist–trajectory curves up to that same moment in the sequence. These visualizations allow the reader to appreciate how the landmark coordinates progress temporally as the signer completes each gesture.

This multi-frame depiction highlights three key aspects of the dataset: (i) the stability of the landmark extraction pipeline across frames, (ii) the dynamic nature of wrist and hand motion for each sign, and (iii) the direct correspondence between the video evidence and the 230-dimensional feature vector used for model training.

An automated workflow was implemented in Python to ensure the homogeneity and quality of the dataset, dividing process into the stages summarized in Table 6.

After completing this process, a database with well-defined technical characteristics was obtained, the specifications of which are summarised in Table 7.

This rigorous standardization and consolidation process is one of the most important methodological bases of the study, allowing us to feed the LSTM and GRU models a clean, balanced, and representative dataset of the natural variations in MSL.

### 3.4. Model Training and Evaluation

As the final stage of the methodology, a rigorous process was carried out for the design, training, and evaluation of RNN models, with the objective of identifying the most effective architecture for automatic MSL interpretation in clinical contexts. The approach focused on architectures such as LSTM and GRU, known for their ability to model sequences and retain long-term contextual information.

In this work, we adopt a pose-based recurrent architecture instead of end-to-end CNN–RNN or Transformer models operating directly on raw video frames. MediaPipe Holistic is first used to extract hand and upper-body landmarks, converting each video into a compact sequence of numerical coordinates. Prior studies have shown that similar MediaPipe–LSTM pipelines achieve strong performance in dynamic sign language recognition while maintaining moderate computational requirements [34]. This skeleton-based representation avoids processing full RGB frames and reduces the amount of training data and GPU resources required compared with image-level CNN–RNN or Vision Transformer models, which are known to be more demanding in these regards [35,36]. Given the pilot-scale nature of this study and the focus on feasibility rather than state-of-the-art benchmarking, recurrent networks (LSTM/GRU) applied to pose landmarks offer a practical balance between temporal modeling capacity, interpretability, and computational efficiency.

A total of 10 experimental configurations were explored, each carefully designed to represent different levels of architectural complexity and computational efficiency. Each configuration was defined by specific hyperparameters such as the number of layers, units per layer, learning rate, batch size, number of epochs, and use of regularization.

To clarify the model selection and tuning procedure, we now explicitly state that the ten evaluated architectures were obtained through structured exploration combining manual selection with a restricted grid over key hyperparameters. More specifically, across both LSTM and GRU variants, the configurations varied in terms of the following: (i) number of recurrent units (64, 128, 256), (ii) depth of the architecture (1, 2, or 3 layers), (iii) learning rates (0.0005, 0.001, 0.002), (iv) batch sizes (32 or 64), and (v) dropout values (0, 0.1, 0.2). All models were trained using the Adam optimizer with standard coefficients $\beta_{1}=0.9$ and $\beta_{2}=0.999$, and Early Stopping monitored the val_accuracy metric with a patience of five epochs. To reinforce reproducibility, all NumPy and TensorFlow random seeds were fixed, and augmented samples derived from the same base video were always grouped within the same split to avoid leakage. The configuration files and training notebooks used in these experiments are available in [37].

Training was performed using TensorFlow (v2.13.0) and PyTorch (v2.3.0), optimized for ARM architecture on a MacBook Pro (Apple Inc., Cupertino, CA, USA) with Apple M3 Pro chip 18 GB of RAM, and macOS Sonoma 14.5. Strategies such as batch normalization, early stopping, dropout, and grid search were implemented to optimize the training time and prevent overfitting.

Table 8 presents a summary of the 10 architectures evaluated in this study, along with some of their corresponding hyperparameters.

To ensure a reproducible and unbiased evaluation, the dataset was divided according to a stratified 80/20 scheme at the sample level, reserving 80% of the sequences for training and 20% for testing. Within the training subset, 10% was internally reserved as a validation subset for model monitoring during training and computing the val_accuracy metric in TensorFlow.

Each sample corresponds to a 30-frame temporal sequence. The split was stratified by class to maintain proportional representation of the 150 signs. All random seeds for NumPy (v1.24.3) and TensorFlow were fixed to guarantee reproducibility. To avoid leakage from synthetic augmentations, all augmented variants derived from the same base video were grouped and assigned to the same split.

Results were recorded per epoch for accuracy (train), val_accuracy (validation), and final precision/recall (test). The categorical cross-entropy (CCE) loss function and the Adaptive Moment Estimation (Adam) optimizer were consistently used across all experiments.

The following expression defines the loss function used:

(2) $$\mathrm{CCE}=-\sum_{i=1}^{N_{C}}y_{i}\cdot \log\left({\hat{y}}_{i}\right)$$

where $y_{i}$ is the true label for class *i*, ${\hat{y}}_{i}$ is the predicted probability, and $N_{C}$ is the total number of classes. We clarify that categorical cross-entropy loss was computed and monitored throughout training and validation for all model configurations. To ensure stable convergence during optimization, loss was also used as the Early Stopping criterion. Because the study focuses on comparative classification performance, metrics such as accuracy, precision, and recall were emphasized in the main results, while loss curves were examined internally to verify proper training behaviour.

This exhaustive procedure enabled the identification of the best-performing models, consolidating a robust and replicable approach for sequence classification tasks in MSL.

## 4. Results and Discussion

### 4.1. Model Performance

To evaluate model performance, ten different LSTM and GRU configurations were implemented and analyzed. These varied in terms of number of layers, units, learning rate, and dropout, and were carefully selected to balance computational complexity and generalization capacity.

**Figure 5:**
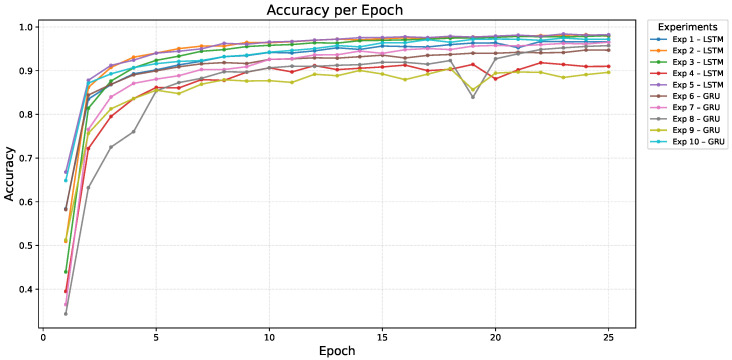
Training accuracy per epoch for the 10 models analyzed. Each line represents a different experiment, grouped by architecture type (LSTM or GRU).

**Figure 6:**
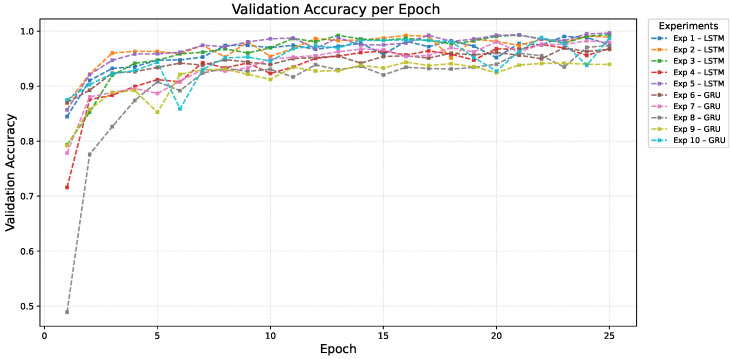
Validation accuracy per epoch for LSTM and GRU models. The curves illustrate generalization behavior during training.

Figure 5 and Figure 6 present the training and validation accuracy curves across 25 epochs, respectively.

Overall, the evaluated models demonstrated consistently high performance. Among them, the LSTM configuration with 128–128 units (Exp. 5) achieved the highest precision on the test set (0.9962) and the best validation accuracy (0.9967), indicating a very low rate of false positives and strong generalization. The deeper LSTM architecture with 128–256–128 units (Exp. 3) obtained a slightly lower precision (0.9941) with comparable validation accuracy, while the more compact LSTM configuration (64–128, Exp. 2) still delivered competitive performance (precision 0.9893). Among the GRU-based models, the 128–128 (Exp. 10) and 64–128 (Exp. 7) configurations reached test precisions of 0.9857 and 0.9822, respectively, remaining close to the LSTM variants. Across all shortlisted models, recall remained high (≥0.96), ensuring that relevant signs were detected consistently. This ranking by precision, summarized in Table 9, reflects the reliability of the proposed recurrent architectures in medical sign recognition, where reducing false positives is a critical requirement. Regularization through dropout and the implementation of early stopping were essential to maintain stability and avoid overfitting during training.

### 4.2. Model Performance Evaluation

The LSTM and GRU models were evaluated using standard metrics for multiclass classification: accuracy, val_accuracy, precision, and recall. These metrics were computed based on the counts of true positives (TP), false positives (FP), true negatives (TN), and false negatives (FN), enabling a comprehensive assessment of both the performance and generalization capability of each model.

(3) $$\mathrm{Precision}=\frac{TP}{TP+FP},\;\mathrm{Recall}=\frac{TP}{TP+FN},\;\mathrm{Accuracy}=\frac{TP+TN}{TP+TN+FP+FN}.$$

### 4.3. Model Ranking and Comparative Analysis

Given the nature of the application—for medical sign interpretation—where the cost of false positives can be high, models were ranked based on their precision. While accuracy (training), validation accuracy (val_accuracy), and recall remain important indicators of generalization and completeness, precision offers a more meaningful signal of trustworthiness in the system’s outputs.

Table 9 presents the top five models ordered by precision. All configurations demonstrate strong performance across metrics; however, precision is used as the primary ranking criterion due to its alignment with the intended use case.

The LSTM configuration with 128–128 units (Exp. 5) achieved the highest precision on the test set (0.9962) and the best validation accuracy (0.9967), indicating a very low rate of false positives and strong generalization. The deeper LSTM architecture with 128–256–128 units (Exp. 3) obtained a slightly lower precision (0.9941) with comparable validation accuracy, while the more compact LSTM configuration (64–128, Exp. 2) still delivered competitive performance (precision 0.9893). Among the GRU-based models, the 128–128 (Exp. 10) and 64–128 (Exp. 7) configurations reached test precisions of 0.9857 and 0.9822, respectively, remaining close to the LSTM variants. Across all shortlisted models, recall remained high (≥0.96), ensuring that relevant signs were consistently detected. These high-performance values reflect the internal consistency of the dataset, as well as the controlled single-signer conditions under which the models were trained. These isolated-sign results are not directly comparable to the narrative-level recall, which reflects a substantially more complex inference task.

Although deeper networks may provide small performance gains, they also entail higher computational costs. In this context, the LSTM (128–128) configuration (Exp. 5) is selected as the primary model, as it offers the best balance between precision, validation accuracy and architectural complexity. The LSTM (64–128) model (Exp. 2) can be adopted when resources are more constrained, whereas the GRU (64–128 and 128–128) configurations (Exp. 7 and Exp. 10) provide faster inference and are attractive alternatives for latency-sensitive applications.

It is noteworthy that validation accuracy exceeded training accuracy in these results. While unusual, this behaviour can occur due to (i) strong regularization (Dropout and L2) penalizing training fit; (ii) differences in data distribution making the validation set slightly less complex; or (iii) early stopping preserving weights at a better generalization point. This observation does not compromise the reliability of the reported rankings but reflects the stability provided by the adopted regularization strategies.

#### Cross-Validation

To further validate the best-performing recurrent architecture identified in the previous experiments, we performed a stratified 5-fold cross-validation. This evaluation step measures the model’s generalization capacity, robustness, and stability across five complementary dataset partitions. For each fold, the selected architecture (the top-ranked LSTM configuration) was independently trained and evaluated, computing accuracy, macro-precision, and macro-recall.

Cross-validation provides a more rigorous assessment than a single train–test split, ensuring that model performance is consistent even when the training and evaluation data vary. This procedure also reveals the variability across folds, helping identify whether the model is stable or sensitive to specific data partitions. A comparison between the 80/20 split and the 5-fold CV is presented in Table 10, where the results confirm that, although the former slightly overestimates performance (as is expected due to the larger training subset), the cross-validation metrics remain consistently high with low variance, validating the robustness of the selected LSTM architecture.

**Figure 7:**
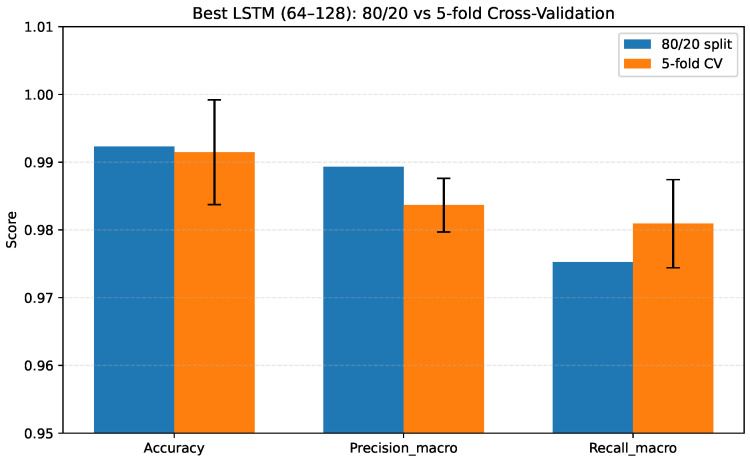
Comparison between 80/20 split and 5-fold cross-validation for the best LSTM architecture. Error bars show standard deviations across folds.

Figure 7 provides a visual comparison between the 80/20 split and the 5–fold cross-validation results, with the former achieving slightly higher scores, as expected (due to the larger training subset). However, the cross-validation bars exhibit small standard deviations (SDs), confirming the selected architecture’s robustness.

**Figure 8:**
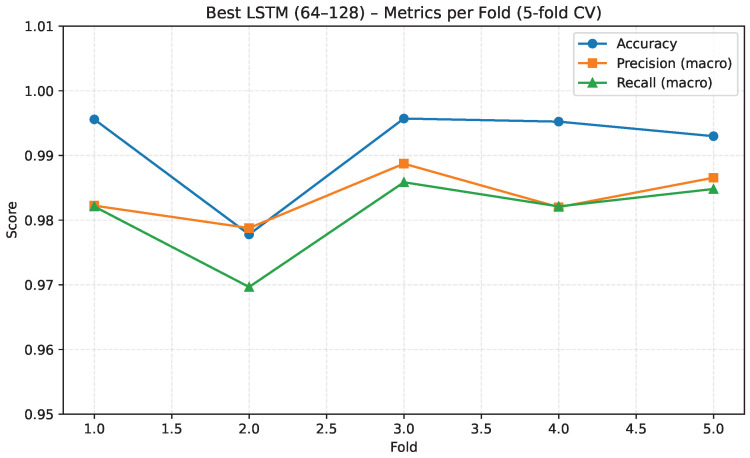
Performance metrics per fold in 5-fold cross-validation for the best LSTM architecture. All folds show consistently high performance.

To further illustrate performance stability across folds, Figure 8 reports accuracy, macro-precision, and macro-recall for each of the five folds. The metrics remain consistently high with minimal variation, demonstrating strong generalization and an absence of fold-specific degradation.

Overall, the cross-validation results indicate that the selected LSTM architecture generalizes well across the evaluated folds, with consistently high performance and low variance. However, these findings require careful consideration because the dataset originates from a single signer, which may introduce subject-specific bias. While the model appears stable across partitions of the available data, further validation with multi-signer datasets is thus necessary to fully confirm its robustness and broader applicability.

## 5. MSL Grammar Inference

This section presents a pilot exploration of narrative-level recognition in MSL. While most existing work focuses on isolated-word classification, real communication requires the ability to interpret sequences of signs that form coherent messages. As an initial step toward this broader objective, we designed an experimental framework that evaluates how a recurrent model behaves when processing short, controlled MSL narratives.

Rather than attempting full linguistic coverage, the study concentrates on a small set of predefined narratives constructed using the curated vocabulary introduced in Step 1 of our methodology. These narratives were collaboratively designed with native MSL users to ensure grammatical plausibility; however, they remain simplified and intentionally constrained in terms of length and complexity. The goal is not to model open-ended discourse, but to analyze whether the system can correctly recover ordered sequences of target signs under continuous signing conditions.

To support reproducibility, each narrative corresponds to a fixed list of expected signs, and the evaluation measures whether the model can infer these signs in the correct temporal order. The six narratives used in this pilot study are summarized in Table 11, which presents the key signs and expected narrative structure.

### 5.1. Sequential Recognition Model and Results

The system uses recurrent neural networks—specifically, the LSTM (64–128) configuration selected in the benchmarking experiments—to process temporal sequences of Holistic keypoints extracted with MediaPipe. Given an input sequence $X=\{x_{1},x_{2},\ldots ,x_{T}\}$, each vector $x_{t}$ contains the 226 spatial features extracted per frame. The model computes

(4) $$h_{t}=f\left(W_{h}h_{t-1}+W_{x}x_{t}+b_{h}\right),\;y_{t}=g\left(W_{y}h_{t}+b_{y}\right),$$

where $h_{t}$ is the hidden state and *f* and *g* correspond to tanh and softmax activations, respectively. This formulation captures the within- and across-sign temporal dependencies required for continuous MSL interpretation.

#### Evaluation Procedure

Each narrative video was processed using a real-time inference pipeline designed to evaluate the model’s ability to recover ordered sequences of signs under continuous signing conditions. The procedure operates frame by frame and applies temporal smoothing, interval segmentation, and dual-metric evaluation.

For each video, frames are read sequentially and analyzed using the Holistic model of MediaPipe with detection and tracking confidence set to 0.85 and 0.25, respectively. The function extract_keypoints_lsm() converts the Holistic output into a 226-dimensional feature vector.

To preserve temporal context, a fixed temporal window of 30 consecutive frames (approximately one second of motion) is maintained. At each step, the LSTM model receives the most recent 30-frame window as input, enabling the continuous modeling of temporal context.

Predictions are generated every two frames (≈15 inferences per second). To reduce instability and suppress spurious detections, a voting mechanism aggregates the last ten predictions:

(5) $${\hat{y}}_{t}=\arg\max_{y}\sum_{i=t-9}^{t}1\left(y_{i}=y\right),$$

and the resulting label is accepted only when its confidence exceeds 0.8. All raw predictions, probabilities, and timestamps are logged for auditability and reproducibility.

For evaluation, each video is partitioned into fixed two-second intervals (60 frames), with each corresponding to one expected target sign from the reference narrative. All predictions falling within an interval are grouped together for interval-level evaluation.

Finally, for each expected sign, two complementary metrics are computed:

- Keyword Detection Recall (KD-Recall): the expected sign is considered detected if it appears at least once anywhere in the entire narrative log.
- Sequential Recall (SR): the sign is counted as correct only if it appears within its corresponding two-second interval, providing a stricter and sequence-aware evaluation.

KD-Recall reflects the model’s raw ability to detect isolated signs during continuous motion, whereas SR provides a more demanding, sequence-sensitive measure assessing temporal alignment throughout the narrative.

To assess the model’s ability to interpret short, continuous signing sequences, we evaluate its performance on six predefined narratives designed using controlled vocabulary and produced by a single signer. Each narrative is segmented into fixed two-second intervals, and the system attempts to recover the expected sign for each interval using the previously described inference pipeline.

The results reported here correspond to SR, which directly reflects the model’s ability to preserve temporal alignment under continuous signing. Table 12 summarizes SR for all narratives. Performance varies across cases, with values ranging from 33% to 62.5% and with the overall sequential recall across all target signs being 45.45%. These results reflect the increased realism and stricter evaluation criteria of the updated methodology; unlike with earlier implementations, a sign is no longer counted as correct if it appears anywhere in the video, but only when predicted within its designated temporal interval. Figure 4 offers a visual reference linking the extracted landmarks with their temporal evolution, providing context for the sequential recall values reported in this section.

**Figure 9:**
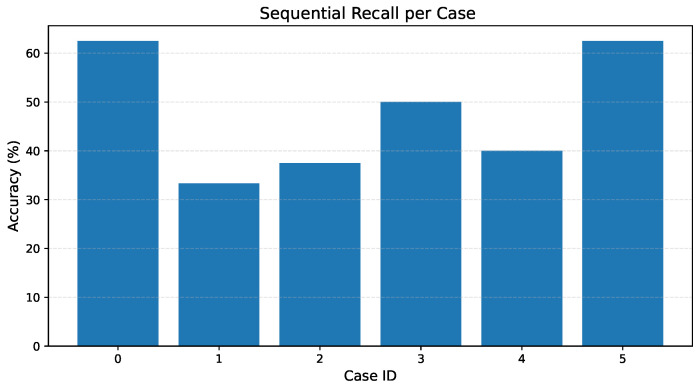
Sequential recall by test case. Performance varies across narratives due to differences in sign transitions and pacing, as well as visual similarity between signs.

Figure 9 provides a visual summary of SR for the six evaluated narratives. Rather than following a monotonic pattern, the results exhibit case-dependent fluctuations: Cases 0 and 5 reach the highest recall values (62.5%), whereas Cases 1 and 3 show lower performance (33.3%) and Cases 2 and 4 fall in the intermediate range (37.5–40%). This variability reflects the influence of narrative structure and sign transitions, as well as each sequence’s intrinsic difficulty.

The higher recall observed in Cases 0 and 5 (both 62.5%) appears to be associated with favorable narrative characteristics rather than an overall improvement in model ability. These cases contain signs that are visually distinct and exhibit clearer temporal boundaries, which facilitates identification within the two-second evaluation intervals.

In contrast, Cases 1 and 3 contain faster transitions, greater co-articulation between signs, and instances of visually similar hand configurations, leading to reduced performance. These observations highlight how narrative difficulty plays a central role in performance variation, underlining SR’s sensitivity to the specific signing patterns present in each case.

**Figure 10:**
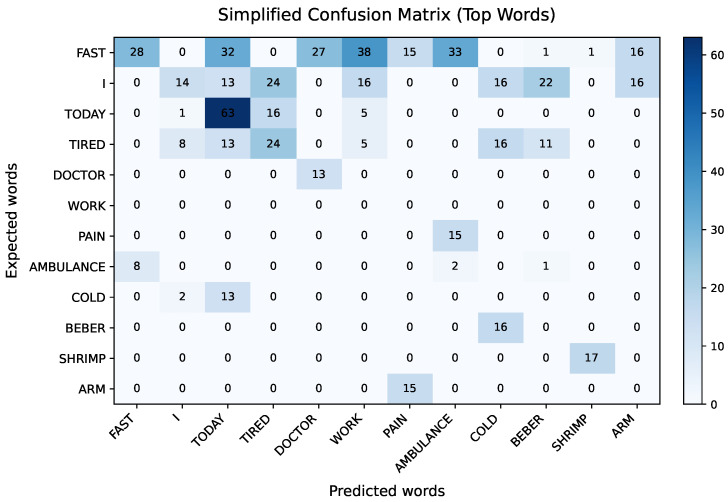
Simplified confusion matrix over the most frequent signs in the narrative experiments. Diagonal cells correspond to correct predictions, while off-diagonal clusters highlight systematic confusions.

To further characterize the types of errors observed during narrative-level inference, a confusion matrix was generated from the interval-level predictions stored in CASES_PERFORMANCE.csv. Only predictions with primary confidence p≥0.80 were considered, and the matrix was restricted to the twelve most frequent signs in the narrative experiments to maintain a readable visualization, as shown in Figure 10.

The confusion matrix reveals several recurrent patterns. First-person and temporal markers (e.g., I, TODAY) are generally recognized correctly; however, they are also confused with each other and with high-frequency context words such as TIRED. Motion-related signs such as FAST and AMBULANCE exhibit notable off-diagonal counts, indicating that rapid hand trajectories in similar spatial regions tend to be misinterpreted. In contrast, some content words, such as SHRIMP or ARM, present stronger diagonal dominance, suggesting that distinctive handshapes and locations are easier to disambiguate.

Overall, this analysis complements the sequential recall results by showing that many errors arise from visually and temporally overlapping articulations in short narratives. These patterns point to the potential benefit of incorporating stronger temporal and linguistic constraints (e.g., sequence or language models at the sentence level) rather than relying solely on frame-level visual similarity.

**Figure 11:**
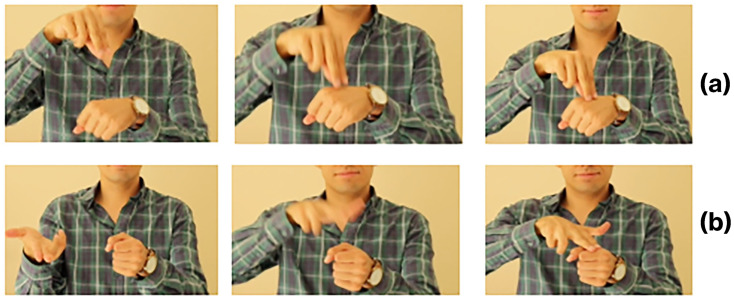
Example of visually similar signs that often cause misclassification. Top row (**a**): frames from the sign DOCTOR. Bottom row (**b**): frames from the sign HOSPITAL.

The analysis reveals frequent confusion between contextually close terms, notably DOCTOR and HOSPITAL. As Figure 11 illustrates, both signs involve similar downward tapping motions on the back of the opposite hand, leading the model to misclassify one for the other. This observation motivates the integration of a sequential language model or attention mechanism to incorporate broader narrative context.

Overall, the narrative-level evaluation confirms that the proposed pipeline can recover portions of short, controlled MSL sequences, demonstrating that sequential interpretation is feasible under constrained conditions. At the same time, the results reveal important limitations: the model was trained on data from a single right-handed signer using one regional variant of MSL, which naturally restricts generalization and increases susceptibility to co-articulation effects, pacing variability, and boundary ambiguity.

Accordingly, these findings should be regarded as an initial exploration rather than a fully developed solution. The framework demonstrates technical viability, but achieving broader and more reliable narrative-level MSL interpretation will require expanded linguistic coverage, multi-signer datasets, and more expressive temporal modeling approaches. This study therefore provides a methodological foundation for future developments.

## 6. Conclusions

This work presents a pilot framework for automatic MSL processing, advancing from isolated sign classification toward the more challenging task of understanding short continuous narratives. Rather than proposing a fully deployable system, the study introduces a methodological and experimental foundation on which future, more comprehensive approaches can be built.

In the first stage, we developed recurrent models (LSTM and GRU) trained on a 150-class dataset enriched through controlled synthetic augmentation. The best LSTM configuration (64–128 units) achieved good performance in isolated-word recognition, with precision and recall consistently above 98%. These results confirm that optimized recurrent architectures are well suited for token-level classification under constrained conditions.

The second stage—narrative-level inference—constitutes the main contribution of this work. Using a reproducible pipeline, each narrative was segmented into fixed temporal intervals and evaluated under strict sequential criteria. The resulting SR (45.45%) reflects the substantial difficulty of recovering ordered sign sequences in continuous motion, where co-articulation, pace variability, and sign-boundary ambiguity strongly affect recognition. Although the model successfully identifies many signs when evaluated in isolation, its temporal alignment degrades as narrative length and complexity increase. These results provide a more realistic view of system performance compared with earlier, overly permissive evaluations, emphasizing the importance of rigorous, sequence-sensitive metrics.

It is important to recognize the limitations of the present work. The dataset was produced by a single right-handed signer using a single regional variant of MSL, which necessarily restricts the model’s generalization and introduces signer-specific bias. While synthetic video augmentation helps to diversify pose and motion patterns, it cannot fully substitute for multi-signer, multi-regional data. The confusion matrix further highlights the impact of visual similarity between signs, suggesting that robust narrative inference will ultimately depend on integrating not only visual models but also linguistic priors capable of capturing MSL grammar and discourse structure.

Despite these constraints, the study offers several substantive contributions: (1) an openly accessible dataset with a standardized preprocessing pipeline, (2) a complete and auditable narrative-level evaluation framework, and (3) empirical evidence demonstrating both the potential and limitations of current recurrent architectures for sequential MSL interpretation.

Future work should explore transformer-based sequence models, signer-adaptive training strategies, multimodal fusion and the integration of linguistic models to enforce temporal and grammatical coherence. Equally important is the collaborative construction of multi-signer datasets to represent MSL’s geographic and stylistic diversity.

In summary, this research should be understood as an initial step toward the long-term goal of developing systems capable of interpreting more complex, naturalistic MSL communication. Furthermore, it provides a transparent experimental baseline and a roadmap for continued progress in the computational study of MSL.

## Figures and Tables

**Table 1:** Comparison of MSL recognition approaches.

Aspect	MX-ITESO-100 (2023) [18]	RNN for MSL (2024) [19]	CNN Finger-Spelling (2024) [20]	RGB-D + RNN (2022) [21]	Our Work
Dataset	100 dynamic signs (verbs, nouns, adjectives, etc.), 5000 videos	20 words (basic vocabulary), 600 clips	21 static signs (dactylological alphabet), 3822 images	30 signs (alphabet, questions, days, phrases), 3000 3D sequences	150 signs (health-oriented), 800 clips per sign
Architecture	CNN + LSTM	LSTM, GRU, Bi-LSTM	CNN + MediaPipe	LSTM/GRU on RGB-D	Optimized LSTM/GRU
Type of Recognition	Static and Dynamic	Dynamic	Static (finger-spelling)	Dynamic	Dynamic
Capture Technique	Depth camera	Camera and web camera	Camera	Depth camera (OAK-D)	FHD camera
Data Augmentation	Not specified	Not applied	Not applied	Gaussian noise	Geom., temporal, lighting
Application	Dataset evaluation	Real-time interpretation	Finger-spelling recognition	3D recognition	MSL in healthcare
Relevant Results	99.12% (validation accuracy)	93% (test accuracy, offline)	83.63% (test accuracy)	97.11% (test accuracy)	99.14% (isolated-sign accuracy)
Global Seq. Recall in Narrative Interpretation	Not applied	Not applied	Not applied	Not applied	45.45%

**Table 2:** Technical and linguistic characteristics of the videos.

Aspect	Specification
File format	MP4, ensuring compatibility and ease of distribution.
High resolution	Full HD (1080p), providing clear visualization of the details of each sign.
Optimal duration	Between 500 and 1200 milliseconds, sufficient to capture the complete movement.
Frame rate	30 frames per second (FPS) for a smooth representation of movements.
Vocabulary coverage	Common, grammatical, familial, temporal, and specialized health-related signs.
Technology used	High-quality camera to ensure precision in capture.
Production	Interpreters and a native MSL teacher, guaranteeing the authenticity and correctness of the signs.

**Table 3:** Summary of basic MSL vocabulary by category.

Category	Quantity	Spanish Vocabulary	English Vocabulary
Basic Signs	6	sí, no, pregunta, duda, bien, mal	yes, no, question, doubt, good, bad
Adjectives	10	duro, suave, normal, frío, caliente, mejor, peor, estresado, rápido, lento	hard, soft, normal, cold, hot, better, worse, stressed, fast, slow
Days of the Week	7	lunes, martes, miércoles, jueves, viernes, sábado, domingo	Monday, Tuesday, Wednesday, Thursday, Friday, Saturday, Sunday
Animals	5	perro, gato, camarón, pollo, abeja	dog, cat, shrimp, chicken, bee
Emotions	2	cansado, confundido	tired, confused
Time	7	ayer, ahora, hoy, mañana, nunca, siempre, diario	yesterday, now, today, tomorrow, never, always, daily
People	6	mamá, papá, esposo, esposa, hijo, hija	mom, dad, husband, wife, son, daughter
Months	12	enero, febrero, marzo, abril, mayo, junio, julio, agosto, septiembre, octubre, noviembre, diciembre	January, February, March, April, May, June, July, August, September, October, November, December
Numbers	10	1, 2, 3, 4, 5, 6, 7, 8, 9, 10	1, 2, 3, 4, 5, 6, 7, 8, 9, 10
Questions	4	cómo, cuántos, para qué, por qué	how, how many, what for, why
Pronouns	1	yo	I
Verbs	14	beber, cocinar, recibir, estudiar, interpretar, ir, no ver, dormir, pelear, trabajar, descansar, comer, correr, caminar	drink, cook, receive, study, interpret, go, not see, sleep, fight, work, rest, eat, run, walk
Medical	47	ambulancia, jarabe, virus, aborto, accidente, hospital, doctor, enfermera, paciente, dolor, enfermo, terapia, pastillas, inyección, contagiar, revisar, calentura, cáncer, infección, infarto, lesión, embarazo, sangre, gripa, garganta, tos, débil, huesos, farmacia, emergencia, inflamación, análisis, coronavirus, cita, fractura, urgencia, orina, popó, mareo, vómito, convulsiones, gases, diarrea, moco, sed	ambulance, syrup, virus, abortion, accident, hospital, doctor, nurse, patient, pain, sick, therapy, pills, injection, infect, check, fever, cancer, infection, heart attack, injury, pregnancy, blood, flu, throat, cough, weak, bones, pharmacy, emergency, inflammation, analysis, coronavirus, appointment, fracture, urgency, urine, stool, dizziness, vomiting, convulsions, gas, diarrhea, mucus, thirst
Body Parts	19	ojo, nariz, oreja, boca, cuello, hombro, espalda, brazo, codo, muñeca, mano, panza, cintura, pene, vagina, piernas, rodilla, tobillo, pie	eye, nose, ear, mouth, neck, shoulder, back, arm, elbow, wrist, hand, belly, waist, penis, vagina, legs, knee, ankle, foot

**Table 4:** Summary of variables by category.

Category	Number of Variables	Variable Range
Video sample	1	1
Classification	1	1
Frame	1	1
Timestamp	1	3
Right Hand	63	4–66
Left Hand	63	67–129
Human Pose	100	130–229
Total	230	

**Table 5:** Video augmentation techniques applied to increase model robustness.

Augmentation	Description
Temporal manipulation	Random speed modification between 85% and 115%, simulating variability in signing fluency.
Rotation	Orientation changes up to $\pm 10^{\circ }$, replicating natural body or camera tilts.
Noise and illumination	Addition of Gaussian noise (mean 0, standard deviation 25), together with brightness and contrast adjustments to emulate different lighting conditions.

**Table 6:** Automated data processing workflow for the construction of the MSL dataset.

Stage	Description
Standardization of headers	A script converted all column headers to uppercase and removed spaces to ensure syntactic consistency and avoid errors during bulk data loading.
Structural validation	Each CSV file was verified to contain the correct number of samples (VIDEO_SAMPLE) per classification, with at least 30 complete frames per sample. Incomplete or inconsistent files were detected, reported, and flagged for removal.
Centralized frame extraction	For each video sample, the sequence was centred by extracting the 30 most representative frames from the middle of the sample, eliminating edges with little data or noise.
Final consolidation	Clean samples were concatenated into a master CSV file, and files were simultaneously generated per classification to maintain individual traceability.
Transformation to numPy format	A module converted each sequence into individual .npy files, storing the 30 frame vectors per sample in structured subdirectories by class and sample number.
Label encoding	A numeric mapping label_map was implemented to associate each word with an integer compatible with multiclass classification tasks.
Data splitting	Training and testing sets were constructed using an 80/20 proportion, ensuring that each sample included the required 30-frame sequences.

**Table 7:** Technical characteristics of the resulting MSL dataset.

Characteristic	Description
Dimensionality per sample	Each sample is represented by 230 variables, including body pose, hand landmarks, and additional metadata.
Temporal structure	Each sequence contains exactly 30 temporal vectors (frames).
Total number of samples	The dataset contains 115,480 processed samples (after filtering incomplete sequences), derived from the original 120,000 intended samples.
Final tensor shape training	115,480×30×226

**Table 8:** RNN architecture configurations evaluated.

Model	Type	Layers (Units)	Learning Rate	Batch	Epochs	Dropout
1	LSTM	[128]	0.001	32	25	–
2	LSTM	[64, 128]	0.0005	64	25	0.2
3	LSTM	[128, 256, 128]	0.0005	32	25	0.2
4	LSTM	[64]	0.002	32	25	–
5	LSTM	[128, 128]	0.001	64	25	0.1
6	GRU	[128]	0.001	32	25	–
7	GRU	[64, 128]	0.0005	64	25	0.2
8	GRU	[128, 256, 128]	0.0005	32	25	0.2
9	GRU	[64]	0.002	32	25	–
10	GRU	[128, 128]	0.001	64	25	0.1

**Table 9:** Top five evaluated models ranked by precision (test set).

Rank	Exp ID	Model	Units	Batch	Precision (Test)	Accuracy (Train)	Val Accuracy (Val)	Recall (Test)
1	3	LSTM	128–256–128	32	0.9957	0.9771	0.9961	0.9887
2	2	LSTM	64–128	64	0.9943	0.9856	0.9954	0.9933
3	7	GRU	64–128	64	0.9939	0.9823	0.9978	0.9869
4	5	LSTM	128–128	64	0.9917	0.9830	0.9952	0.9910
5	1	LSTM	128	32	0.9901	0.9677	0.9930	0.9872

**Table 10:** Comparison between single 80/20 split and 5-fold cross-validation for the best LSTM architecture.

Setting	Accuracy	Precision (Macro)	Recall (Macro)	Accuracy SD	Precision SD	Recall SD
Single 80/20 split (best LSTM)	0.996709	0.996164	0.991689	–	–	–
5–fold cross-validation (best LSTM)	0.991462	0.983664	0.980916	0.007723	0.003960	0.006506

**Table 11:** Test cases used for model evaluation.

Case	Vocabulary (Spanish)	Vocabulary (English)	MSL Narrative (Spanish)	MSL Narrative (English)
0	Ayer, lunes, yo, beber, frío, hoy, yo, cansado	Yesterday, Monday, I, drink, cold, today, I, tired	AYER LUNES YO BEBER FRÍO, HOY YO CANSADO	YESTERDAY MONDAY I DRINK COLD, TODAY I TIRED
1	Hoy, parque, perro, morder, brazo, yo, dolor, ambulancia, venir, rápido	Today, park, dog, bite, arm, I, pain, ambulance, come, fast	HOY PERRO PELEAR, YO BRAZO DOLOR, AMBULANCIA RÁPIDO	TODAY DOG FIGHT, I ARM PAIN, AMBULANCE FAST
2	Ahora, yo, mal, mareo, dolor, hospital, ir, doctor, ver	Now, I, bad, dizziness, pain, hospital, go, doctor, see	AHORA YO MAL, MAREO, DOLOR, HOSPITAL IR, DOCTOR	NOW I BAD, DIZZINESS, PAIN, HOSPITAL GO, DOCTOR
3	Hoy, restaurante, camarón, comer, yo, mal, emergencia, llamar	Today, restaurant, shrimp, eat, I, bad, emergency, call	HOY RESTAURANTE CAMARÓN COMER, YO MAL, EMERGENCIA	TODAY RESTAURANT SHRIMP EAT, I BAD, EMERGENCY
4	Hoy, esposa, convulsiones, ambulancia, rápido	Today, wife, convulsions, ambulance, fast	HOY ESPOSA CONVULSIONES, AMBULANCIA RÁPIDO	TODAY WIFE CONVULSIONS, AMBULANCE FAST
5	Hoy, trabajo, yo, mano, infección, doctor, ver, rápido	Today, work, I, hand, infection, doctor, see, fast	HOY TRABAJO YO MANO INFECCIÓN, DOCTOR VER RÁPIDO	TODAY WORK I HAND INFECTION, DOCTOR SEE FAST

**Table 12:** Sequential recall by test case.

Case	Recall (%)
0	62.50
1	33.33
2	37.50
3	33.33
4	40.00
5	62.50
Global Recall	45.45

## Data Availability

The dataset supporting the findings of this study is publicly available on Zenodo [33].
